# Multi-layered e-feedback anxiety: An action research study among Chinese learners using peer feedback activities in an academic writing course

**DOI:** 10.3389/fpsyg.2023.1062517

**Published:** 2023-03-02

**Authors:** Sijia Xue, Yanchao Yang, Jianxia Du, Fangtong Liu

**Affiliations:** ^1^College of Teacher Education, Southwest University, Chongqing, China; ^2^Faculty of Education, University of Macau, Taipa, China; ^3^Qinggong College, North China University of Science and Technology, Tangshan, Hebei, China; ^4^School of Foreign Languages, Shanxi University, Datong, China

**Keywords:** e-feedback, learning anxiety, educational technology, mobile learning, academic writing

## Abstract

There is a gradual increase in the use of e-feedback in higher education, but issues regarding learners’ anxiety remain unresolved. In light of the learners’ anxiety, e-feedback would essentially become a formality if they are not proactive in providing constructive feedback. This action research examines three cycles of e-feedback activities performed by 12 doctoral students in an academic writing course in a public university in Macau, China. Specifically, the e-feedback activity involved a comprehensive use of various new educational technology tools, namely Moodle, WeChat and Rain Classroom. This study reveals that the causes of students’ anxiety when using e-feedback are multi-layered, mainly from the use of smartphones as a communication medium for conducting formal learning activities and the lack of interpersonal and English skills for conveying their thoughts when providing e-feedback. The traditional Chinese culture about the importance of “face” and interpersonal harmony also has impacts on learners’ e-feedback delivery. These findings shed new lights on pedagogical practice in higher education.

## 1. Introduction

There has been a proliferation of research on feedback in recent years, focusing on its influence on the development of student writing (Lee, 2017; Hyland and Hyland, 2019). Meanwhile, in academic writing classes, peer feedback, a popular learning approach incorporated in process-based, student-centered, and scaffolded learning, has evolved as a popular pedagogical method and has received increasing attention from researchers and practitioners (Xu and Liu, 2010). Student peer feedback offers several educational benefits for academic writing, for instance, it improves students’ communicative capabilities, facilitates meaningful interactions among peers, greatly engages students in learning, and offers new perspectives on writing (Huisman et al., 2018; Man et al., 2018).

With the trend of integrating technology into education, the format of peer feedback has been diversified (Stapleton and Radia, 2010). There is a variety of new technologies available today that allow for text, audio, and video feedback, which have not only had a profound effect on the nature of peer feedback activity, but also on the quality of students’ learning experiences (Cunningham, 2019; Killingback et al., 2019). Furthermore, the popularity of smartphone use in learning also promotes the development of technology-assisted peer feedback (hereafter “e-feedback”) due to its flexibility in time and space as well as its user-friendliness (Alrasheedi and Capretz, 2015). However, despite the potential benefits of e-feedback on both feedback givers and receivers (Nicol et al., 2014; Papadopoulos et al., 2017), it also faces challenges in many aspects. One of them is learners’ anxiety about e-feedback activities in L2 learning.

Previous research has demonstrated the negative relationship between learner anxiety and learning-related performances (von der Embse et al., 2018). The studies focusing on e-feedback anxiety are scarce in comparison to other anxiety research fields, such as foreign language anxiety, computer or mobile phone anxiety, test anxiety. In particular, there is a lack of qualitative research exploring the factors contributing to learners’ anxiety regarding e-feedback. Without this knowledge, it is difficult for L2 practitioners to design and implement e-feedback activities that can effectively realize the intended pedagogical purposes. To fill in this gap and solve the corresponding educational problems, the current study explores how technological, social, and cultural factors may lead to anxiety among Chinese PhD students when using e-feedback in an L2 academic writing course.

Feedback cannot be conducted in an educational environment without involving emotion (Hadden and Frisby, 2019). The act of providing feedback is inherently face-threatening (Witt and Kerssen-Griep, 2011; Brummernhenrich and Jucks, 2016; Hadden and Frisby, 2019). There is no doubt that negative feedback may pose a threat to the rapport between feedback givers and recipients. Hence, maintaining a positive relationship between feedback providers and recipients is challenging, especially when feedback is rigidly demanded (Hadden and Frisby, 2019). In addition, Mikulincer (1988) presented abundant evidences suggesting that recipients of negative feedback eventually abandon the task due to learned helplessness. There has been considerable emphasis placed on the need to mitigate face threats in feedback settings by instructional feedback scholars (Trees et al., 2009). Therefore, the results of the study are of importance in that they can provide guidance for instructors to apply instructional feedback strategies to minimize negative student emotional reactions, and to engage students to actively provide feedback to students’ writing assignment *via* e-feedback channel, which may protect their students’ face and reduce the feedback anxiety. Thus, the study’s findings can be used to guide instructors on how to minimize negative emotional reactions in students by applying instructional feedback strategies, and by encouraging students to actively provide feedback *via* e-feedback channels, thereby protecting their face and reducing feedback anxiety for their students. What’s more, this study is of importance by identifying potential sources of factors in relation to e-feedback anxiety in academic writing. Last, in light of the growing importance of technology in the writing classroom, the current study investigated how e-feedback anxiety might be mitigated through the use of diverse technology-enhanced tools in online instruction, for instance, the use of emoji and the option of anonymous peer feedback especially in light of the global COVID-19 pandemic, under which opportunities for implementing online education at a large scale have been provided (Xie et al., 2020).

## 2. Literature review

### 2.1. Theoretical framework

#### 2.1.1. Common sources of anxiety in educational contexts

One of the most investigated situation-specific categories of anxiety in education is test anxiety. Based on the trait–state anxiety theory (Spielberger, 1972), Spielberger and Vagg (1995) proposed the transactional process model for test anxiety. This theoretical model shows a dynamic process of anxiety in assessment or test situations. In an evaluation context, individuals with sufficient skills (learning and test taking skills) may regard the situation as less threatening. The negatively appraised or reappraised test situation (within one’s intrapersonal process) may in turn evoke cognitive and affective reactions including worry and emotionality which may result in irrelevant thoughts or behaviors to the task. This model illustrates the complex mechanism of anxiety of individuals in evaluation situations, which is common in education activities (such as teacher’s evaluation and peer feedback), suggesting that both learning skills and intrapersonal characteristics of a learner could be linked with test anxiety in a loop relationship.

Foreign language anxiety (FLA) is another widely investigated situation-specific anxiety in education (Horwitz et al., 1986; MacIntyre, 1999; Aydin, 2008). Horwitz et al. (1986) first adopted the Foreign Language Classroom Anxiety Scale (FLCAS) and evaluated FLA in three aspects: communication apprehension, test anxiety and fear of negative evaluation. Communication apprehension includes social communication and foreign language proficiency (Horwitz et al., 1986). These three main components of FLA in class can be a useful framework for investigating anxiety in class situations when communication, test and evaluation occur frequently. It indicates that students’ communication apprehension skills, ability to manage test anxiety and fear of negative evaluation are important concerns in foreign language learning contexts.

In sum, in educational settings, students could be confronted with test anxiety in test/evaluation situations or FLA in learning contexts using foreign languages. Theoretical models of test anxiety and FLA suggest that students’ anxiety in learning activities might be associated with study skills, trait, apprehension ability, social communication skill and foreign language proficiency. For students in e-learning activities receiving e-feedback, in a foreign language learning context, it seems clear and important to consider test anxiety and FLA since the learning context contains both evaluation and foreign language.

#### 2.1.2. Control-value theory of academic emotions

Pekrun (2006) proposed the control-value theory of academic emotions, which explained the reciprocal links between academic emotions (e.g., anxiety, boredom), antecedents/predictors (environment and appraisal) and effects/consequences (learning and achievement). This theoretical model can be a suitable framework for studies investigating academic/achievement-related emotions in learning activities. Pekrun et al. (2011) measured academic/achievement emotions in three academic situations (class, learning and test), including enjoyment, hope, pride, relief, anger, anxiety, shame, hopelessness, and boredom. In the control-value theory, achievement emotions are influenced by individual’s emotion regulation and cognition regulation (appraisals), including “control” (e.g., expectations for success) and “value” (e.g., perceived importance) of achievement activities and outcomes. Anxiety is an emotion with negative (failure) value and medium control (Pekrun, 2006). In the reciprocal relationships, one’s cognition regulation (control and value) can be affected by emotion, motivation, learning strategies, and the environment (e.g., achievement feedback). Thus, it seems that the mechanism of academic emotion could be understood as a circle of interactive relationships between emotion, appraisal (control and value), learning and the environment. Academic emotions (e.g., anxiety) might be lined with complex factors, including personal appraisals and environmental factors (e.g., feedback). This theoretical model clearly points out the reciprocal link between learning environment and academic emotions, for instance, between e-feedback situation and anxiety.

### 2.2. Empirical studies

#### 2.2.1. E-feedback in learning

Empirical studies have proved the benefits of e-feedback in learning (Tuzi, 2004; Lu and Bol, 2007; Krause et al., 2009; McCabe et al., 2011; Ciftci and Kocoglu, 2012). Some studies proved the benefits of teachers’ e-feedback or computer released feedback (Krause et al., 2009; McCabe et al., 2011). Online peer feedback was also demonstrated to be conducive to learners (Tuzi, 2004; Ciftci and Kocoglu, 2012; Hwang et al., 2018). Lu and Bol (2007) found that anonymous peer e-feedback was more beneficial than identifiable peer feedback. It shows the potential limitation or negative effects of identifiable peer e-feedback. However, few studies focus on the potential negative effect of peer feedback. One study investigated online peer feedback among English-as-a-second-language (ESL) students and found both positive and negative effects (Guardado and Shi, 2007). The students reported low self-confidence in peer commenting and communication was not always effective. In all, current studies proved that e-feedback can help students, especially in English learning contexts. However, limitations and shortcomings of e-feedback need to be further investigated.

#### 2.2.2. Social anxiety and peer feedback

Social anxiety seems to be an important concern in cooperative learning which requires much social communication. The delivery and reception of peer feedback can arouse an intense and persistent fear of social occasions and/or performance situation (Cartney, 2014), what Rachman termed as “social anxiety” (Rachman, 2013, p. 171). It has been frequently investigated in education especially in cooperative learning situations. Students were frequently reported to adopt coping strategies including safety behaviors (minimizing conspicuousness) and avoidance of learning activities (Russell and Topham, 2012). Studies also identified students’ experiences of social conflicts or unsafety during cooperative learning (Raes et al., 2015). Low psychological safe and poor interpersonal skills may result in negative emotions toward feedback and anxiety (Slof et al., 2016; Frazier et al., 2017). In addition, personality can play a significant role in cooperative learning, since introverted students may have greater difficulty interacting and learning from others (Lee and Lee, 2006; Banaruee et al., 2017). There is evidence that social anxiety is a common phenomenon in education, particularly in cooperative learning activities where peer feedback is required. Students with social communication difficulties might suffer psychological distress when learning with others and receiving feedback, evaluation or social pressure. Therefore, students’ difficulties in different cooperative learning contexts, such as anxiety toward e-feedback, must be explored further.

Several studies identified the links between social anxiety and technology use (Caplan, 2007; Liu and Kuo, 2007; Ha et al., 2008). Among 343 American undergraduates, Caplan (2007) found that social anxiety significantly predicted preference for online social interaction. Liu and Kuo (2007) revealed that Internet addiction was significantly predicted by social anxiety and low interpersonal relationship among 555 Taiwan students. Ha et al. (2008) reported that Korean adolescents who used mobile phones excessively experienced higher social anxiety. These studies indicate that students’ use of technology (either Internet or phones) has potential links with social anxiety or their social interaction skills. Thus, it is possible for students to feel anxious about their interpersonal interactions online when engaging in technology-based learning activities. Furthermore, the presence of social anxiety remains an open question in cooperative smartphone-based learning situations (learning with online peer feedback), when social communication is frequent through the use of a smartphone.

#### 2.2.3. Foreign language anxiety and peer feedback

Empirical studies of peer feedback have largely focused on foreign language learning situations, such as peer feedback in second language writing (Yu and Lee, 2016). Foreign language anxiety (FLA) in feedback-driven learning seems to be an important concern. Several empirical studies have explored FLA in different contexts and found similar correlations (Kitano, 2001; Wang, 2009; Mak, 2011). It has been determined that the main factors related to FLA are fear of negative evaluation/fear of failure, as well as low self-perceived proficiency in foreign languages. In an online cooperative learning situation, however, it remains unclear whether fear of negative evaluation becomes more serious or not. Additionally, it is unclear whether foreign language learning in that new environment with online social communication can lead to increased anxiety in students with foreign language difficulties. Thus, more studies are needed to explore the possible risk factors in an online cooperative (feedback-driven) foreign language learning context.

#### 2.2.4. Cultural influence on learning and anxiety

Students’ cultural background can affect their willingness to participate in peer feedback activities (Soden, 2013). It seems important to consider cultural influence on e-learning activities especially with e-feedback in cooperative situations. Although cooperative learning has been widely practiced in different countries, the effects or usefulness seems to be unstable in different cultural backgrounds (Sharan, 2010). Cooperative learning, especially feedback driven learning, benefits students in learning and social communication (Law, 2008; Tarhan et al., 2013; Booysen and Grosser, 2014). However, students also experienced difficulties in getting familiar with the instructions of cooperative learning and had trouble with getting involved in groups (Ciftci and Kocoglu, 2012; Tarhan et al., 2013; Booysen and Grosser, 2014). In a study in Vietnam, Nguyen-Phuong-Mai et al. (2012) found that culture was a barrier of implementing cooperative learning. The traditional Vietnamese cooperative methods were preferred, and western cooperative learning could not be applied smoothly. Thus, cooperative learning methods may not be successful in different contexts or cultures. The degree to which students accept peer feedback in their learning may be influenced by cultural differences.

Chinese culture has often been reported as a culture of anxiety, as Hu (1944) argues that Confucius culture is about the concept of “face.” Based on that, it seems possible that Chinese students grown up in Confucius culture might experience difficulties in cooperative learning since they tend to avoid losing “face” or keep safe as discussed previously. Wang (2009) reported that the Taiwan students with Chinese culture background experienced anxiety caused by cultural difference when learning abroad. However, Yang (2018) reported that British undergraduates perceived significant higher levels of academic anxiety than Chinese students, while the reasons might be cultural differences or other environmental factors (e.g., educational experience). It is interesting that the empirical finding contradicts with the previous claim that Chinese students are not as anxious as British students. This clearly indicates the need to further explore whether culture plays an important role in students’ anxiety. Thus, the cultural influence can be complex in different contexts. It seems necessary to investigate whether culture impacts students’ anxiety in peer feedback learning activities, especially the Chinese students from a more introverted culture.

### 2.3. Research aims

In summary, the feeling of anxiety during peer feedback activities seems to be a topic that needs more investigation. Since cooperative learning requires communication skills and inevitable evaluation process, whether test anxiety or social anxiety exists or not remains unknown. In foreign language learning contexts, fear of negative evaluation and social anxiety, are the possible risk factors for students. It also seems necessary to explore the possible anxious feelings during foreign language cooperative learning contexts. A recent empirical study confirmed the control-value theory of achievement emotions in a computer-based cooperative learning context (Camacho-Morles et al., 2019). Three achievement emotions (enjoyment, anger and boredom) were clearly identified in computer-based cooperative learning. However, no studies have investigated anxiety in a technology-based cooperative learning context. Thus, this study aims to explore how factors related to technology, individual capacity (social and language), and culture affect online peer feedback among Chinese learners.

## 3. Method

### 3.1. Action research and participants

We took the qualitative hermeneutic action research approach which emphasizes participation by the researcher to try out new strategies and to evaluate the outcomes (Johnson and Christensen, 2017). Action research was described by Lewin (1948) as a process consists of four iterative stages: planning, acting, observing, and reflecting. In action research, the focus is on developing, implementing, and evaluating plans for improving practice through action or intervention in a spiral of research cycles (Kemmis, 1982). Action research offers many benefits for educators committed to a critical, investigative process of improving school practice, policy, or culture. First, action research provides the researcher with the opportunity to work on a problem, not only providing answers to the problem but also contributing to the development of theory (Johnson, 2012). A further benefit of this approach is that it empowers participants, enables change, and facilitates the development of organizational learning (Fueyo and Koorland, 1997; Meyer, 2000). What’s more, as the project progresses, modifications can be easily implemented (Koshy et al., 2010). Therefore, action research method was adopted for the current study. The high involvements of action researchers allow them to plan their practices and to improve cycles of action based on observations and reflections with rigorous research documentation.

The current study involves a 12-week academic writing course conducted in a public university in Macau, China, where we carefully planned our course design and conducted three cycles of e-feedback to examine leaners’ anxiety. Our principal investigator is the instructor of the course and another two researchers, as teaching assistants of this course, were also actively involved in classroom interactions and group activities to closely examine students’ emotional status. Twelve PhD students enrolled in this course and we included all the students as our participants. Of the participants, eight are female and four are male, and their age ranged from 24 to 41 years (*M* = 29.6). Pseudonyms were given to students to protect participants’ identity information. As shown in Table 1, their educational backgrounds varied among domains of education, psychology and business administration. Prior to entering the doctoral study, they worked and lived in different provinces and cities all around China. Though we followed the convenience sampling approach, these doctoral students with their characteristics in terms of geographical, academic and English backgrounds could somehow represent the general crowd of doctoral students.

**Table 1:** Participants’ information.

Group	Name	Gender	Age	Hometown	Educational Backgrounds
1	Melody	Female	29	Sichuan	Business English & Education
	Katy	Female	24	Macau	Educational Psychology
	Skyler	Male	25	Zhuhai	Physics Teaching
2	Stella	Female	35	Tianjin	Science Education
	April	Female	26	Hefei	Preschool Education
	Jessica	Female	25	Datong	Physical Education
3	Jay	Male	31	Hunan	TESOL
	Lily	Female	25	Qinghai	English Teaching
	Sherry	Female	30	Beijing	Literature and Art
4	Bobbie	Female	41	Foshan	Marketing Management
	Josepher	Male	30	Jiangxi	Pedagogy
	Bill	Male	34	Macau	Business Administration

Prior to our study, the instructor reached consent with the students about data collection during the course period and these participant students were allowed to withdraw from the study at any point of the course. In the first week class, the instructor divided all the students into four groups based on their English proficiency, academic backgrounds and research experience.

### 3.2. Course background and research procedure

**Figure 1:**
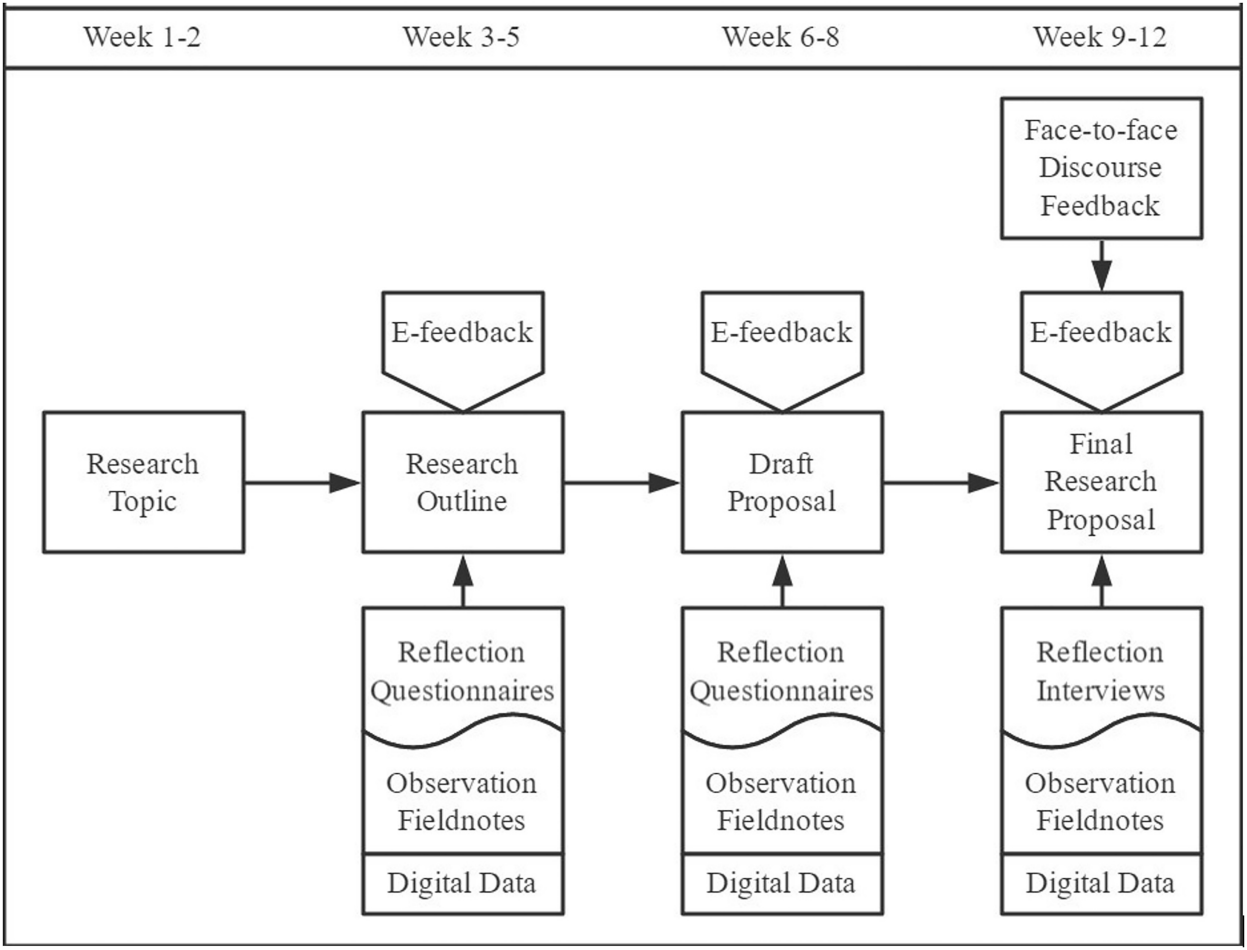
Course procedure and data collection.

The aim of this doctoral course is to equip students with necessary academic writing skills in writing a group research proposal including three sections, namely introduction, literature review and methodology. Figure 1 illustrates the course procedure of teaching students to write a research proposal with four phases, namely developing research topic, writing research outline, writing draft proposal and writing final research proposal.

Based on the procedure of conducting action research, the current study initiated the first stage, Plan, by identifying the practical and pertinent problem and proposing some possible solutions. During the second stage, Act, the principal investigator implemented the plan. Next, in the following stage, Observe, the co-authors collected data and observed the effects of the implemented plan. In the final stage, Reflect, all authors decided what needed to be changed or improved based on the outcomes, then revised or modified it for the next step. This action research cycle was repeated three times to address the issue.

Data for this study were drawn from open-ended self-reported questionnaires, interviews, observation, and self-emerging digital data, which were used to triangulate with other sources of information. Specifically, we conducted three e-feedback sections during three phases of proposal writing. Detailed schedule of questionnaires, interviews and observation are also shown in the figure. Meanwhile, self-emerging digital data provided by the technological platforms were also collected. We added a face-to-face oral feedback as a complementary peer feedback section in the final cycle of e-feedback based on the reflections of the prior two cycles, and the reason for this will be further discussed in the results section. In the open-ended self-reported questionnaires for the first two cycles and the final reflection interview, students’ responses to the e-feedback anxiety were collected, which was used to triangulate with the observation. We transcribed and analyzed the interview data using a qualitative inductive approach (Strauss and Corbin, 1998). For the first step, researchers carefully examined the transcripts in order to identify the common themes associated with e-feedback anxiety. A second aspect of the analysis is to triangulate the results of the interviews with classroom observation notes and generated feedback contents pertaining to the research question. This will provide a different perspective on the participants’ e-feedback anxiety and how it has evolved over time. Data analysis results were continuously sent to participants for their comments, which were taken into account for data interpretation.

Our e-feedback involves comprehensive applications of Rain Classroom, WeChat, Moodle. Moodle^1^ in our course was for students to submit their assignments and then students could post peer feedback in the platform in a formal but asynchronous way. WeChat^2^ peer feedback was used in our course as an informal way of communication to instantly share information and provide feedbacks among peers. Rain Classroom^3^ in our course was used when a group presented their writing assignments. Students from other groups could post instant peer feedback through the Rain Classroom platform while listening to the presentation. Table 2 introduced the general information of these three types of technological platforms and their unique characteristics in conducting peer feedback.

**Table 2:** General information of e-feedback (Rain Classroom, WeChat, Moodle).

Types of feedback	Developer	Main application	Characteristics of feedback
Moodle	Martin Dougiamas	Learning Management Platform	The comments are more formally structured.
			Students express thoughts and ideas in a relatively long paragraph.
WeChat	Tencent (China)	Social Networking APP	The comments are informal and might contain other unrelated information.
			Free expression helps facilitate the conversation and hence generates more information benefiting learning.
Rain Classroom	Tsinghua University	Presentation Toolkit	The comments specifically focus on students’ presentation of their writing.
			The comments from peers are more accurate and concise.

## 4. Results

### 4.1. Cycle 1: initiating e-feedback (week 3–5)

#### 4.1.1. Planning and action

Our aim in the first cycle of e-feedback was to get students involved in successfully utilizing all the three platforms and achieving specific peer feedback tasks on generating the research outline. There were no guidelines or restrictions in the first cycle because we wanted to observe students’ anxiety in an e-feedback setting that is as natural as possible.

#### 4.1.2. Observation

From our self-emerging digital data, we collected 31 comments on Moodle, 142 on WeChat and 65 on Rain Classroom. As expected, the students were curious about the way of using different types of technological platform to conduct peer feedback due to its novelty. However, when it came to the formal peer feedback, students hesitated to type their thoughts. Only those who were more confident in their English language proficiency would type a long paragraph on the Moodle platform. Students communicated better on WeChat because most of the students usually use it for social networking. Though their WeChat conversations contain lots of unrelated wordings, students were more relaxed and more willing to share their views. Finally, in our Rain Classroom peer feedback section, their pressure increased when students were watching presentation and posting feedback simultaneously. Data in the Rain Classroom platforms showed that some students just gave up formulating comments.

#### 4.1.3. Reflection

In the later open-ended self-reported questionnaires, students’ responses were consistent to what we observed. Specifically, three types of anxiety, namely English anxiety, smartphone anxiety and communication anxiety, were revealed in the cycle of e-feedback. Skyler’s previous academic background is physics teaching. Although he has basic communication skills in English, typing English within a short time using technology brought him pressure. He recalled his anxiety in terms of language:

*I really did not have much self-confidence because I realized that my English proficiency was relatively low among the classmates. So, I think this weakness impeded me to communicate smoothly with others, making me a little inferior and always nervous during the peer feedback sections in class.*

As understanding others’ presentation was also part of the learning task, the multitasking of comprehension, evaluation and providing feedback was a huge challenge to the students. Bill felt anxious when using smartphone to type Rain Classroom feedback:

*We have to spend time typing our opinions timely when the presentation is processing. My typing speed is slow, so I may miss some contents of the presentation when I type.*

Jessica, a girl who values the Chinese traditional culture of harmonious communication, noticed that when one provided feedback to others, his or her ID appeared on the screen. Her anxiety came from her worry about maintaining a good interpersonal relationship with peers:

*As the feedback is given on Moodle, WeChat and Rain Classroom with givers’ ID, everyone knows who is giving the feedback to whom. Thus, I was worried because conflict might be created.*

Overall, the first cycle attained the goal of getting students to use e-feedback despite that the content of their feedback seemed too general and cautious. We noticed that students’ anxiety was also caused by their unfamiliarity with the technology used and we assumed that students would be more adapted to e-feedback in the next cycle. Thus, we made adjustments in our second cycle, trying to address the issues.

### 4.2. Cycle 2: Repeating e-feedback (week 6–8)

#### 4.2.1. Planning and action

Providing the viability of conducting e-feedback through multiple technological tools, the second cycle focused on guiding students to post more comments on peers’ draft proposal and to expand peer learning effectiveness. We encouraged students to post at least three comments regarding various aspects of academic research in each activity. Other settings of e-feedback remained the same as in the previous cycle.

#### 4.2.2. Observation

**Figure 2:**
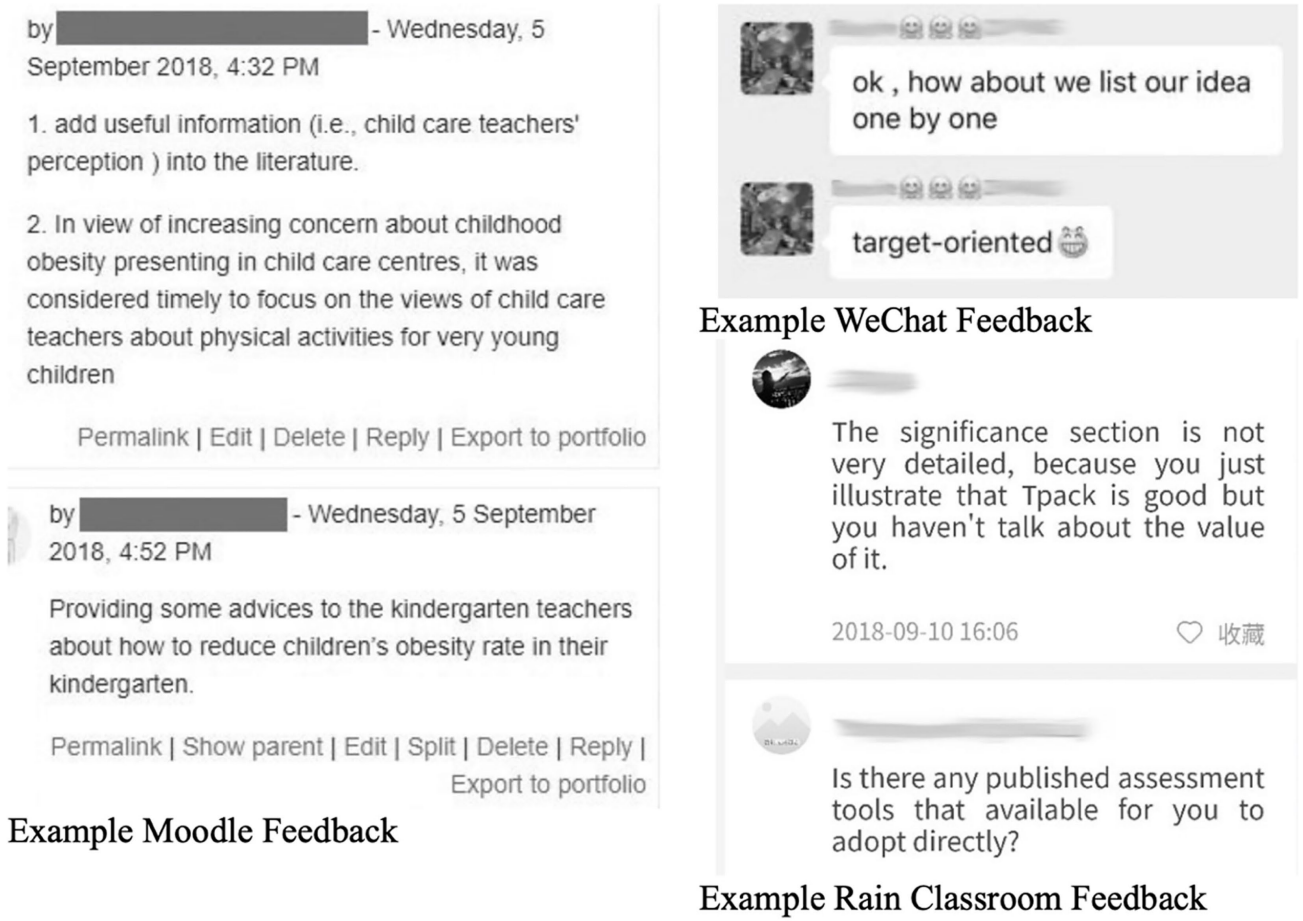
Examples of e-feedback.

From our self-emerging digital data, we witnessed a slight increase of feedback collected (43, 154, 82 comments on Moodle, WeChat, Rain Classroom, respectively). In terms of the content of feedback, students posted more content-oriented comments. Figure 2 demonstrates some examples of e-feedback.

From the feedback collected from Moodle and WeChat, students tended to concentrate more on specific problems of peers’ writing assignments rather than general issues, which was a good sign of improvement. We also noticed that when one group of students saw the Rain Classroom feedback posted on the screen, it seemed that they were eager to defend for themselves. We quickly moved on to next group’s presentation since class time is limited as scheduled previously. But we felt that we might need to reserve time for student discussion in the future.

#### 4.2.3. Reflection

In the follow-up open-ended questionnaires, although some students still expressed their anxiety in using English, the issues related to smartphone improved as the participants became more used to the application of these feedback tools. For example, April noted:

*I’m getting more used to the technology and it is more comfortable to use rain classroom rather than giving oral feedback face to face, because it allows us more space to think about other’s writing work and give more well-organized feedback on it.*

However, participants still expressed their negative views toward e-feedback. They claimed that they would try to defend their opinions when receiving others’ comments. E-feedback was not an effective way to do this and often led to further misunderstanding. Stella pointed out that e-feedback did not allow them to undertake thorough discussion with peers:

*I still prefer face-to-face talk about our writing work because more substantive information can be delivered, and I can argue with my peers if I do not agree with the feedback.*

Communication anxiety is still a major concern for our Chinese participants, several students agreed:

*In China, we value the way of polite communication. But sometimes when we conduct communication activities through online software, like WeChat, Rain Classroom, it would cause some minor conflicts. Because in the virtual world, we can only see the cold words. The words can be understood in many different ways and cause misunderstandings easily.*

One student, Bill, suggested an option to improve this situation:

*If there is an anonymous option to hide our names, then maybe you can propose some negative comments, right?*

At this point, we realized that adjustments of our e-feedback activities were needed. Otherwise, the participant students would still be trapped in the zone of worry and uncertainty that impede the effectiveness of e-feedback peer learning. We noticed that some students had already tried to use emoji to improve the awkwardness of providing harsh e-feedback. Inspired by students’ creative use of emoji, subsequent actions were taken in the third cycle.

### 4.3. Cycle 3: Reforming e-feedback (week 9–12)

#### 4.3.1. Planning and action

Drawing on learning from the previous two cycles, we refined and improved our e-feedback activities. Trying to alleviate their worries about interpersonal communications on Moodle and WeChat, we shared some examples of using emoji, polite wordings and affective language to ease harsh communication. Another significant reform is to add a follow-up face-to-face section after the Rain Classroom feedback for students to further discuss the comments among peers. We also turned on the anonymous function in the Rain Classroom system so that students would dare to express their opinions directly when they noticed any shortcomings of the presentation.

#### 4.3.2. Observation

In the third cycle, students posted much more feedback than the previous two cycles, shown in Table 3. From our observation, students no longer hesitated to express their thoughts and views because they knew that emoji, polite wordings and affective language would help prevent unnecessary conflicts.

**Table 3:** E-feedback collected in three cycles.

Cycle stage	Writing assignments	Moodle	WeChat	Rain Classroom
1st Cycle	Research outline	31	142	65
2nd Cycle	Proposal draft	43	154	82
3rd Cycle	Final proposal	62	217	97

**Figure 3:**
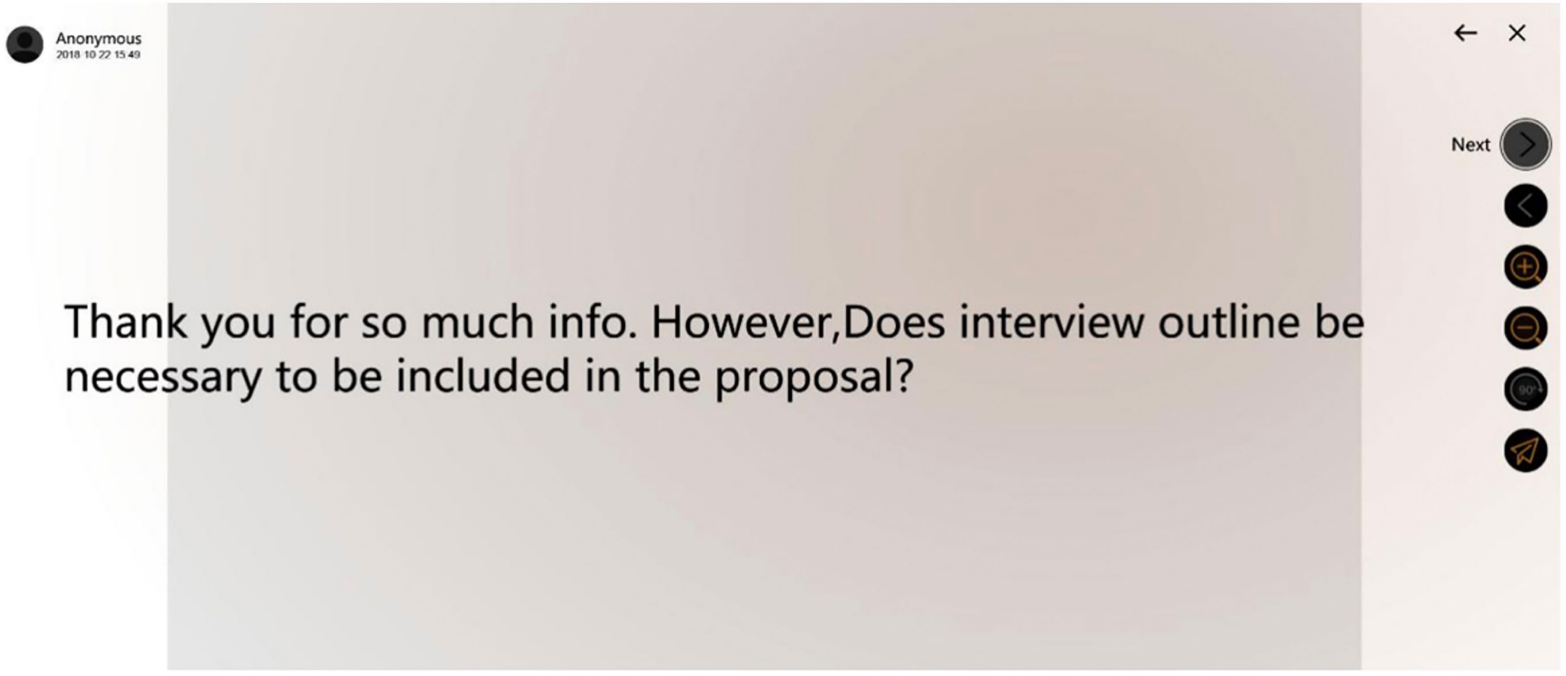
Examples of anonymous Rain Classroom feedback.

Furthermore, the follow-up oral feedback enabled students to achieve higher-order communication. The anonymous function also helped reduce personal factors and the discussion was more content-oriented. Figure 3 is an example of anonymous Rain Classroom feedback posted on the screen after one group’s presentation.

#### 4.3.3. Reflection

After the three cycles of e-feedback, we had another reflection interview with our students to inquire about their anxiety issues. Firstly, they explained that their anxiety toward English language and smartphone use gradually alleviated as they practiced e-feedback repeatedly. Secondly, with the guidance form the instructor on interpersonal communications skills of using emoji, polite wordings and affective language, students’ communication anxiety was largely eliminated and even result in happy moments of peer learning experience because of using funny emoji. For instance, Lily emphasized her preference toward emoji:

*I am very fond of using emoji. Actually, emoji has already become a must for me when chatting with others online. The funny and rich contents in emoji are good icebreaker at very beginning under any situations online. And the most updating emoji can shorten the distance between each other, letting us know that we are all young people.*

Jessica’s cultural stereotype turned communication anxiety into a pleasant experience, as she claimed:

*Engaging in such a harmonious and enjoyable atmosphere with my peers and listening to the suggestions they raised for me, I felt it was a fascinating process to improve my work.*

Moreover, Melody pointed out that the follow-up face-to-face feedback greatly enhanced the effectiveness of peer feedback. It adds supplementary effects of deeper communication on the basis of the portable, instant, communicative features in e-feedback. She recalled a moment of her presentation:

*Once one of my peers commented on my theoretical framework in Rain Classroom by pointing out “the logic and associations among each variable are not clear enough.” Her comment actually identified the weakest part which I am least confident with in my research proposal. I valued this piece of feedback and felt eager to hear more opinions from my peer. In the subsequent discussion session, I proposed that I would like to reexamine the feedback related to my theoretical framework and was warmly responded in the discussion.*

Although there were still rooms for improvement to establish a better learning environment for e-feedback, various types of anxiety identified in the first cycle of the current study were resolved to some extent. Further implications would be discussed in the next section.

## 5. Discussion and implications

### 5.1. Influences from smartphone use to e-feedback anxiety

Our study found that students’ anxiety increased when they used smartphones as a communication medium for e-feedback. The reasons are two-folded. On the one hand, smartphone itself causes anxiety when students used it casually in social communication. Students are always keeping a close eye on their smartphones because their fear of missing out important messages and always need for reassurance. As a result, poor self-regulation is reported by smartphone learners as they might distract from class when they take out their smartphones (Yang et al., 2018). In our study, students were required to type e-feedback through the Rain Classroom platform. During our classroom observation, we noticed that students switched from the Rain Classroom platform to their frequent use apps from time to time, which is an important signal of their struggle of concentrated learning. The transfer from a casual device to a formal serious teaching and learning tool caused greater pressure to students, which affected their peer feedback activities and consequently resulted in poorer learning outcomes.

On the other hand, students’ e-feedback activities using smartphones would bring about anxiety because e-feedback is closely bound to their assignments and course achievements. These e-feedback activities were targeted on specific learning tasks including the outline writing, first draft writing and final proposal writing of the academic writing. When students embraced a learning task or an assignment which would subtly influence their course achievement, they would encounter huge burden in terms of deciding adequate feedback they should provide. Therefore, they might hesitate when typing their comments through the Rain Classroom platform thinking about their comments might affect others’ grades. Just as one of our participants noted that whether her peers found her feedback useful or not would affect how she posted feedback in the next round. In essence, e-text peer feedback using smartphones is far more complicated than pure individual learning. As a result, anxiety might be triggered unexpectedly in this form of peer learning.

This finding has important implications for teaching pedagogy with ICT. Firstly, in today’s classroom setting in higher education, it is impossible to ask students not to use smartphones. Educators have gradually realized the inevitability of using students’ own devices (BYOD) in the reconstruction of teaching process (Lu et al., 2018). Although the use of e-feedback brought anxiety to students, in the meantime, students started to notice that smartphones as a learning tool also require concentration which would reduce their problematic smartphone use to some extent. Secondly, e-feedback on smartphone is a new learning practice which takes time for students to get used to. Teachers might have some hands-on practice on giving and receiving messages or emoji through virtual learning platform such as Rain Classroom. This classroom activities motivated students to try out as many functions as they can to get familiar with the learning platform before getting to the more formal e-feedback section. Finally, teachers should ensure that the learning objectives as well as the evaluation standard of the e-feedback activities are communicated explicitly to students. The evaluation standard should focus more on encouraging students to actively involve in peer feedback activity rather than merely assess their contents of comments. This would help set up clear learning strategies for students to conduct e-feedback and reduce their worries about grades.

### 5.2. Influences from individual capacity to e-feedback anxiety

E-feedback anxiety becomes multi-layered and more serious when feedback givers lack interpersonal and English skills. First, since peer feedback is a social activity, learners with weak interpersonal or communicative skills are more likely to experience anxiety in peer feedback activities. When working with peers, learners are exposed to interpersonal conflicts such as psychological unsafety and trusting relationship building (Raes et al., 2015). For learners who cannot properly apply interpersonal skills to deal with these barriers, they may gradually accumulate negative emotion and resistant attitudes toward giving and receiving feedback (Slof et al., 2016). Particularly, if they do not feel psychologically safe in the group environment, their anxiety level increases (Frazier et al., 2017). This is especially true for introverted learners who tend to experience more psychological costs during social interaction in e-feedback activities. One possible explanation is that those with introvert personality are shy to provide e-feedback. This echoes with previous research findings where students in introverted groups posted less messages and had less social, interactive, and cognitive interactions than those in the mixed groups and extroverted groups in the online discussion (Lee and Lee, 2006). Meanwhile, when introverted learners receive e-feedback that contains criticism, it is easier for them to experience negative emotion before they accept the feedback. This is to some extent in line with previous findings that introverted learners were less receptive toward and benefited less from explicit corrective e-feedback than their extroverted peers (Banaruee et al., 2017).

In addition, using English as the medium for peer feedback activity, some learners who have low English proficiency or who are not confident with their English capacity tend to experience foreign language anxiety and be more anxious in giving feedback than those who are more competent and confident about their English. Students in this course come from different educational backgrounds. Their major disciplines in bachelor’s and master’s degrees are diverse, including English, science education, physic education, early childhood education, psychology, culture, and management. Over half of them did not have experiences about English peer feedback before they attended this course. When they used a less familiar language to write e-feedback in a designated short time, their technology anxiety and foreign language writing anxiety co-appeared and made their total anxiety level increase. Particularly, when students saw some of their peers could do this smoothly, their peer pressure and lack of language proficiency further increased their stress worry about their performance.

These observations imply that in order to lower down students’ multi-layered anxiety caused by technology use, lack of interpersonal skills and incapability of foreign language use, teachers can also take actions to nurture students’ communicative and linguistic skills. In this way, learners are supposed to feel more comfortable and confident in using a foreign language to deliver e-feedback and actively applying social skills in learning through social interactions. Meanwhile, they can guide students not to put e-feedback as a formal learning activity that requires their formal language use, but as a relaxing, comfortable, and enjoyable one with the communication skills and ways they adopt in their daily life. One example is the encouragement of students’ use of emoji when giving e-feedback, which has been found to be beneficial to students’ positive emotion by increasing the enjoyment, playfulness and pleasure and decreasing the information processing loads (Thompson et al., 2016; Hsieh and Tseng, 2017; Duan et al., 2018). This further enhances learners’ acceptance toward both the e-feedback activity and e-feedback received from peers (Zumbrunn et al., 2016). Besides, when such familiar daily usage is transferred to learning contexts, it is conducive for students to decrease the anxiety level caused by academic social interaction.

### 5.3. Influences from culture to e-feedback anxiety

Despite anxiety brought by individual interpersonal and linguistic skills, the Chinese culture of “face” and harmony shared by these students also causes learners’ e-feedback anxiety. In order not to embarrass others’ feelings and to protect themselves, many Chinese learners think twice and act cautiously when they want to post negative feedback. Such cognitive loads brought by cultural values increased the anxiety level of students when they gave e-feedback. Specifically, one core concept of this Confucius-heritage culture is the concept of face (Hu, 1944). Losing face in front of others is one of the top things Chinese would like to avoid. In the e-feedback contexts, signs of losing face in front of the peers include receiving negative feedback, giving feedback that is not considered as valuable, exposing their lack of content knowledge in front of others *via* giving feedback, and making linguistic mistakes in giving feedback. Furthermore, creating circumstances that cause other people to lose face is also considered as a selfish and disrespectful behavior (Bond, 1996). Based on this value, some students are reluctant to give explicit negative feedback to their peers. If they think of a negative point that can help peers to make progress, they may feel anxious and would struggle to provide this feedback.

This perspective indicates that considering the potential benefits of e-feedback activities in promoting students’ knowledge and language skills, teachers are advised to build up the awareness of training Chinese learners how to make better use of peer feedback. When the cultural value, such as the concept of face in China, becomes a barrier to effectively realizing the benefits of a learning approach (i.e., e-feedback), teachers may rethink if this value should be weakened to some extent during the application of e-feedback activities. In this way, students’ e-feedback anxiety brought by their own culture can possibly be lessened, hence conducive to their learning *via* e-feedback activities.

## Data availability statement

The raw data supporting the conclusions of this article will be made available by the authors, without undue reservation.

## Ethics statement

The studies involving human participants were reviewed and approved by the Research Ethics Committee at the University Macau. The ethics committee waived the requirement of written informed consent for participation.

## Author contributions

JD conceived and designed the analysis and conducted the instruction of the course academic writing. FL collected the data, performed the analysis, and drafted manuscript preparation. SX revised it critically for important intellectual content. YY conducted transcribed the data collected and conducted substantive translation. All authors reviewed the results and approved the final version of the manuscript.

## Funding

This work was funded by the Chongqing Social Sciences Foundation, China (Grant No. 2022NDYB131).

## Conflict of interest

The authors declare that the research was conducted in the absence of any commercial or financial relationships that could be construed as a potential conflict of interest.

## Publisher’s note

All claims expressed in this article are solely those of the authors and do not necessarily represent those of their affiliated organizations, or those of the publisher, the editors and the reviewers. Any product that may be evaluated in this article, or claim that may be made by its manufacturer, is not guaranteed or endorsed by the publisher.

## Footnotes

^1^Moodle is a free open source learning platform created by Martin Dougiamas and now is supported by over 90 certified Moodle Partners around the world. It can share class materials and host online forum discussion. Official Website of Moodle: https://moodle.com/.

^2^WeChat is one of the most popular social medium software in China. Official Website of WeChat: https://www.wechat.com/en/.

^3^Rain Classroom, developed by Tsinghua University, is a free teaching toolkit built on PowerPoint. It aims to enhance the interactivity of classroom teaching when using PowerPoint presentation and to emerge digital tracing data of students’ learning activities simultaneously. Official Website of Rain Classroom: https://www.yuketang.cn/.
